# Evaluating the impact of field epidemiology training programs: a descriptive review of the published literature

**DOI:** 10.1186/s12960-025-01015-1

**Published:** 2025-08-27

**Authors:** James A. Flint, Tambri Housen, Rachel Hammersley-Mather, Martyn D. Kirk, David N. Durrheim

**Affiliations:** 1 University of Newcastle, Newcastle, Australia; 2 Australian National University, Canberra, Australia

**Keywords:** Field epidemiology training, Evaluation, Impact

## Abstract

Field epidemiology training programs (FETPs) are designed to equip public health professionals with the skills necessary to investigate, monitor, and respond to disease outbreaks and other public health emergencies. Since the 1950s, when the first FETP started in the United States, the training model has been adopted by numerous countries around the world. Today, there are 98 FETPs in operation, and over 20,000 graduates. This review assesses published studies that report on the evaluation of FETPs. A literature search yielded 402 records, with 16 publications meeting inclusion criteria after duplicate removal and eligibility screening. The 16 FETP evaluations encompassed 37 national and four regional assessments across 26 countries. Most of the evaluations were descriptive reviews using quantitative methods focusing on outputs and short- or medium-term outcomes. Only four published evaluations focused on longer term impacts of an FETP. The evaluations describe and quantify numerous outputs and outcomes, providing evidence of trainees and graduates applying skills to strengthen core health system functions. Several challenges were also identified, including poor utilisation of FETP graduates by senior management stemming from a limited understanding of what field epidemiologists can contribute to the health system. While these evaluations indicate that FETPs are successful training programs, there are relatively few published impact evaluations providing the level of evidence increasingly expected by funders and stakeholders. There is a need and opportunity to develop tools and resources to support FETP evaluators in the implementation of impact evaluations.

## Introduction

Since their inception in 1951, field epidemiology training programs (FETPs) have been a cornerstone of public health preparedness, equipping over 22,000 graduates to tackle diverse disease threats worldwide [1]. As health security concerns have intensified, FETPs have become increasingly relevant, playing a vital role in national, regional, and global preparedness and response. Their role is recognized by the most recent version of the International Health Regulations (IHR), which sets targets for field epidemiology training [2]. Similarly, the Global Health Security Agenda, which launched in 2014 to support IHR implementation, recognised FETPs as an important element in strengthening health security [3]. The recent COVID-19 pandemic further highlighted the importance of FETPs and prompted renewed calls for significant investment in this workforce. A former United States Centers for Disease Control and Prevention (CDC) Director recently stated that FETPs may be the single most important thing CDC contributes to global health, advocating for a massive scaling up of FETPs, and highlighting their crucial role in building a more resilient global health architecture for the next pandemic [4].

The first FETP, known as the United States Epidemic Intelligence Service (EIS), adopted a training model that focused on *learning while doing,* which combined the hands-on experience of medical residency with the case study methodology used by the Johns Hopkins School of Hygiene and Public Health. Training through service became the distinguishing feature of this and future FETPs [5]. In 1975, the first FETP outside the United States was established in Canada; in 1980, the first FETP outside North America was established in Thailand [6, 7]. Subsequently, additional programs were established in Asia, the Americas, Australia, Europe, and Africa [8]. Today, there are 98 FETPs serving more than 200 countries throughout the world [1].

The work-integrated learning model of FETPs differentiates them from other higher learning opportunities. Despite the program adaptations and variations, FETPs share the following core principles: (i) programs are *field-based,* adopting a work-integrated learning model where fellows spend 20–25% of their time in the classroom and 75–80% in the field [7, 9], (ii) programs are *competency-based*, focusing on developing technical knowledge and skills, rather than achieving academic milestones [10], and (iii) fellows participate in *work-integrated learning* while placed in public health departments and agencies. With guided mentoring and supervision, fellows analyse surveillance data, detect and respond to disease outbreaks and other health emergencies, conduct epidemiological studies of interest to host agencies, communicate scientific findings, and translate those findings into public health actions [7].

With time, FETPs adapted as they responded to country needs and demands. Curricula were tailored to address specific cultural contexts, disease priorities, and public health systems. Some programs partnered with degree-granting academic institutions [11]. Others incorporated a laboratory component and became known as Field Epidemiology and Laboratory Training Programs (FELTPs) [12–16]. Some adopted a regional approach and supported the training needs of several countries. Examples of this include the Central America FETP, French-speaking East African FETP, and Central Asia FETP [1, 7, 16, 17]. An increasing number of One Health and veterinary-based FETPs were also developed [18, 19]. Other specialisations offered by FETPs included management and leadership, monitoring and evaluation, social and public health sciences, health inspection and health education [18, 20].

To train field epidemiologists across different levels of the health system and with diverse educational backgrounds, a three-tiered training approach was adopted by many countries [17]. These tiers are often referred to as frontline (or Basic)—3 months in duration, intermediate—6–12 months in duration, and advanced FETP—a 2-year program [21, 22]. In 1992, the Public Health Schools Without Walls program started. This program emphasized the combination of rigorous academic and extensively supervised practical experience [23]. Some countries in Africa developed FETPs under the umbrella of the Public Health Schools Without Walls, such as Zimbabwe in 1993, Uganda in 1994, and Ghana in 1995 [24]. These programs differ from the more traditional FETPs in that fellows spend more time in the classroom (~ 40%) and a Master of Public Health is awarded to graduating fellows.

The Training Programs in Epidemiology and Public Health Interventions Networks (TEPHINET) was founded in 1997 to act as a global coordinating body and facilitate exchange of knowledge and best practices [25]. To support program consistency and quality, TEPHINET developed a continuous quality improvement handbook for FETPs [26]. In 2016 TEPHINET introduced a formal accreditation process and published a program evaluation guide, further promoting program consistency and quality [26, 27]. To date, 18 programs have been accredited [28]. Whilst there are relatively few published evaluations of FETPs, there is growing interest in assessing program outcomes and impacts.

This review identifies and assesses published studies that report on evaluations of FETPs. It describes FETP evaluations conducted worldwide and examines the evaluation models and approaches used to evaluate the effectiveness of these training programs. While it summarises key outputs, outcomes, and impacts of the reported in the evaluations, this review does not systematically assess or draw conclusions about the outcomes and impact of FETPs, either individually or collectively.

## Methods

This descriptive review identified potentially relevant documents by searching the PubMed, Directory of Open Access Journals (DOAJ), and Scopus databases. There was no time limitation on the search period; all papers published up to 31 December 2022 were included. Language was restricted to English. Search terms were combined using Boolean operators and adapted for each of the electronic databases. The search terms and strategy are shared in Appendix A.

Papers were included in the review if they focused on the evaluation of an FETP or applied epidemiology training program, regardless of the specific nature of the program. This included frontline, intermediate, and advanced programs, as well as programs with a specialisation. Quantitative, qualitative, and mixed-method studies were all included. Papers were excluded if they assessed a specific training program output or outcome rather than evaluating the whole program. Papers that listed or described outputs or outcomes of an FETP without a clearly defined evaluation methodology were excluded. Conference abstracts, seminar reports, and non-English publications were excluded.

The final search results were exported into EndNote, with duplicates removed. We screened the titles and abstracts of all identified studies against the inclusion and exclusion criteria. Papers clearly not meeting the selection criteria were discarded. The remaining publications were assessed by reviewing the full text of the article to determine the final selection. Reference lists of the final selected articles were reviewed to identify any additional relevant studies. FETPs were grouped based on the country’s income level, as defined by the World Bank [29].

## Results

**Fig. 1:**
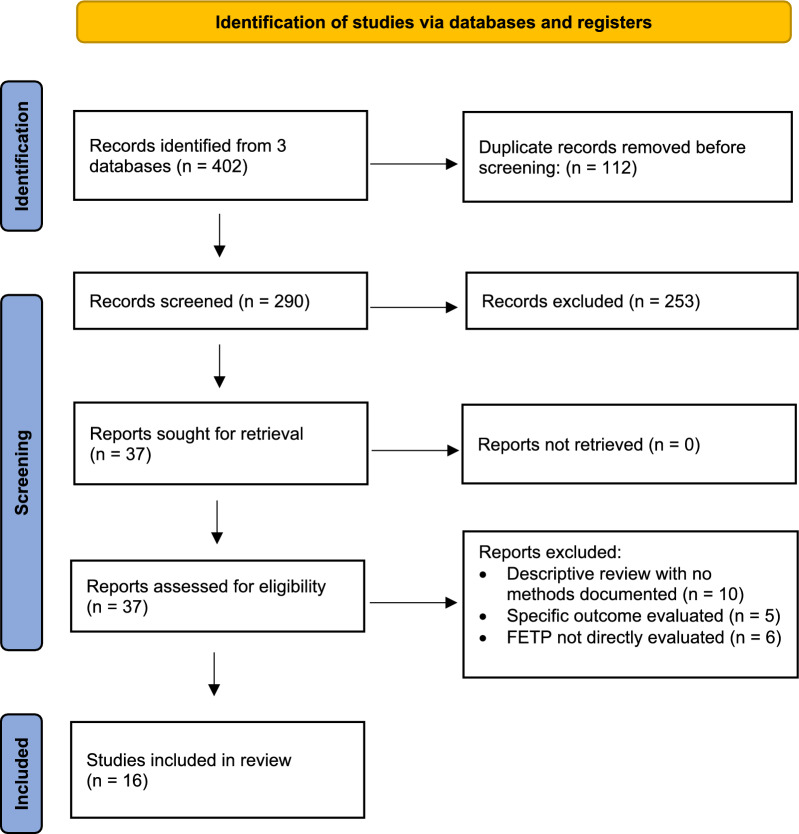
Summary of search results and record screening

Figure 1 outlines the process used for selecting papers for inclusion in the review. A total of 402 records from electronic databases were identified. After removing duplicates and screening for eligibility, a total of 16 publications were included in the final full-text review.

### Description of FETP evaluations

The 16 published FETP evaluations included 37 national program evaluations from 26 countries and four regional program evaluations. Most programs evaluated were advanced FETPs (n = 36, 88%); there were also four frontline FETPs (10%) and one Intermediate FETP (2%). Evaluations ranged in publication date from 2003 to 2022. An overview of the evaluations is provided in Table 1.

**Table 1:** Summary of published field epidemiology training program evaluations, 2003–2022

Author, year	Country (Income level)	FETP Type^d^	Specific evaluation objective or key evaluation questions	Evaluation design	Data collection methods	Analysis	Evaluation level^e^
Pappaioanou et al. [40], 2003	United States (High)	Advanced	To describe program outputs and outcomes and the professional experiences of veterinarians who have graduated from EIS from 1951–2002.	Descriptive, with case study	▪ Document review	Quantitative	▪ Output ▪ Outcome
Moolenaar et al. [43], 2004	United States (High)	Advanced	Evaluated two specific outcomes of field epidemiology training—publications and career choices, for EIS classes of 1991–1996.	Descriptive	▪ Document review ▪ Existing data	Quantitative	▪ Output ▪ Outcome
Thacker et al. [44], 2011	United States (High)	Advanced	Described the first 60 years of field epidemiology deployments (Epi-Aids), highlighting the breadth of health problems addressed, evolution of methodologies, scope of activities and impact of investigations on population health	Descriptive	▪ Document review	Quantitative	▪ Output ▪ Outcome ▪ Impact
Bhatnagar et al. [34], 2012	India (Lower Middle)	Advanced (degree granting)	To evaluate the first seven years of the Chennai FETP to identify strengths and weaknesses.	Descriptive—Theory based	▪ Document review ▪ Survey – graduates	Predominantly Quantitative	▪ Output ▪ Outcome
Dick et al. [42], 2014	United States (High)	Advanced	Evaluated outcomes of the first nine classes of alumni with particular attention to how the fellowship affected alumni careers, mentors’ careers, host site agency capacity, and competencies of the applied epidemiology workforce.	Descriptive	▪ Review of existing data ▪ Survey – graduates ▪ Survey – mentors	Mixed methods	▪ Output ▪ Outcome ▪ Impact
Volkov et al. [35], 2014	Multi-country: Africa, Asia, Central America and Europe^c^ (Low, Lower Middle, Upper Middle)	Advanced (including laboratory track, degree granting, national and regional)	Evaluated the quality of conference abstracts submitted by participants of FETPs to assess how well trainees applied knowledge and skills gained in training	Descriptive, consensus expert review	▪ Document review	Quantitative	▪ Output
Lee et al. [37], 2017	Korea (High)	Advanced	Evaluated the field epidemiology system that had been in place in South Korea for 16 years since 1999 and to suggest appropriate future improvements to this system.	Descriptive	▪ Tests – fellows ▪ Survey – fellows	Quantitative	▪ Outcome
Gatei et al. [14], 2018	Multi-country: Africa and Europe^b^ (Low, Upper Middle)	Advanced (laboratory track)	Sought to understand why several FELTPs had discontinued the L-Track and list the major achievements of the FELTP.	Descriptive	▪ Survey – FELTP directors	Predominantly Quantitative	▪ Output
Dey et al. [30], 2019	United Kingdom (High)	Advanced	To ascertain whether the UK FETP met its objectives to (i) strengthen capacity and provide national epidemiology services, (ii) develop a network of highly skilled field epidemiologists with a shared sense of purpose working to common standards, and (iii) raise the profile of field epidemiology by embedding it into everyday health protection practice.	Descriptive - Kirkpatrick’s Model (Level 3 and 4)	▪ Focus groups - supervisors and staff at training sites ▪ Interviews – stakeholders ▪ Survey – graduates and fellows	Predominantly Qualitative	▪ Outcome ▪ Impact
Macharia et al. [39], 2020	Kenya (Lower Middle)	Frontline	Evaluated the impact of Frontline graduates on disease surveillance completeness and reporting timeliness.	Ecological	▪ Existing data	Quantitative	▪ Outcome
Al Nsour, et al. [31], 2021	Multi-country Eastern Mediterranean^a^ (Low, Lower Middle, Upper Middle, High)	Advanced	To evaluate (i) the degree to which trainees applied what they learned during training when they were back on the job, and (ii) the degree to which targeted outcomes occurred as a result of the training (targeted outcomes included improved data collection on reportable diseases, improved outbreak investigation and response, improved surveillance systems and surveillance reports, improved health policies).	Descriptive - Kirkpatrick’s Model (Level 3 & 4)	▪ Survey –graduates ▪ Survey –technical advisors	Predominantly Quantitative	▪ Outcome ▪ Impact
Roka et al. [32], 2021	Kenya (Lower Middle)	Frontline	Measured the effect of Frontline FETP on participant workplace practices regarding quality and consistency of public health data, critical interaction with public health data and improvements in on-time reporting.	Descriptive, repeated measures - Kirkpatrick’s model (Level 3 & 4)	▪ Survey–graduates ▪ Interview–graduates	Mixed Methods	▪ Output ▪ Outcome
Wilson et al. [36], 2021	Tanzania (Lower Middle)	Intermediate	Evaluated the outcomes of a new intermediate FETP by assessing improvement in trainees’ epidemiological knowledge, skills and capacities at the workplace using data from 2017 to 2020	Descriptive	▪ Tests –fellows ▪ Survey –fellows ▪ Interview –fellows	Predominantly Quantitative	▪ Output ▪ Outcome
Buffington et al. [41], 2022	United States (High)	Advanced	Evaluated the participation of nonmedical scientists in the EIS program and their subsequent employment	Descriptive	▪ Document review	Quantitative	▪ Output
Collins et al. [45], 2022	Guinea (Low)	Frontline	Described the characteristics of graduates; how participating in the Frontline FETP was perceived by trainees and their supervisors, and how it improved the quality of their work. The evaluation also investigated the availability of key surveillance and reporting tools associated with surveillance strengthening efforts.	Descriptive	▪ Document review ▪ Interview –graduates ▪ Interview –supervisors and directors at training sites	Predominantly Qualitative	▪ Outcome
Kebebew et al. [38], 2022	Ethiopia (Low)	Frontline	Assessed surveillance-related knowledge, skills, and performance among trained and untrained officers. Evaluated the impact of FETP-Frontline amongst district surveillance officers in Ethiopia.	Comparative cross-sectional	▪ Survey –graduates and controls	Quantitative	▪ Outcome

^a^Saudi Arabia, Egypt, Jordan, Pakistan, Iraq, Morocco, Yemen and Sudan

^b^Kenya, Ghana, Nigeria, Tanzania, Armenia/Azerbaijan/Georgia, Kazakhstan, Cameroon, Mozambique, Rwanda and Mali

^c^China, Ethiopia, Kenya, Nigeria, Pakistan, South Africa, and Vietnam and 3 regional FETPs (Central America, Central Asia, and South Caucasus)

^d^Frontline FETPs were defined as any basic FETP running for 3 months; intermediate FETPs were defined as FETPs running for between 9-12 months; Advanced FETPs were defined as any FETP of 2 years or more

^e^For the purposes of this review, outputs are defined as products, projects or activities which result from the program; outcomes are short and medium term effects of the program; and impacts are long-term effects of the program

Programs from the United States and Kenya were featured in five and four evaluations, respectively. In the United States, the EIS program was evaluated on four separate occasions and the Applied Epidemiology Fellowship was evaluated once. In Kenya, the Advanced FETP and Frontline FETP were both evaluated twice. There were two evaluations from Ethiopia, Nigeria, Tanzania, Pakistan, and the South Caucasus Region. Tanzania was the only country where an Intermediate FETP was evaluated. The 26 country programs evaluated cover all six of the World Health Organization regions (Europe, South-East Asia, Western Pacific, Eastern Mediterranean, Africa, and Americas). FETPs from high- (n = 8), upper-middle- (n = 4), lower-middle- (n = 17), and low-income (n = 8) countries were represented.

### Evaluation models and approaches

Most of the evaluations were descriptive (n = 14, 88%); three were based on the Kirkpatrick model [30–32], specifically levels 3 and 4 (Kirkpatrick level 3 measures behavioural changes in the workplace after the training, while level 4 focuses on whether the anticipated outcomes were achieved after the training program [33]). One of these evaluations was a repeated measures study [32], which collected data at graduation (baseline) and intervals of six, twelve and, eighteen months post-graduation. One of the descriptive evaluations was theory-based with the researchers referencing the programs’ logic model as a basis of the evaluation [34]. The two evaluations that were not descriptive used comparative cross-sectional and ecological study designs. The comparative cross-sectional study compared the outputs and outcomes of FETP graduates to untrained surveillance officers working at the same level and in similar roles to the graduates. The untrained surveillance officers served as a counterfactual (control) group. The ecological study used spatial analysis to assess an association between the completeness and timeliness of surveillance reporting across the country from 2014 to 2017 and the number and location of FETP graduates.

The stated evaluation objectives were often quite general, such as identifying strengths and weaknesses of the FETP [34], assessing the application of knowledge [31, 35, 36], describing program outcomes [36], or making recommendations to strengthen the program [37]. Some were more targeted, seeking to understand why a specific FETP model had been discontinued [14] or assessing the impact of the training on improving surveillance [31, 32, 38, 39], outbreak response [31] or health policies [31]. Two evaluations focused on reviewing the outcomes of the FETP by tracking specific cohorts of veterinarians [40] and nonmedical scientists [41] graduating from EIS. In addition to assessing the impact on graduates, one evaluation sought to assess the effect of the FETP on the sites hosting the fellows during their training [42]. Other evaluation objectives focused on publication outputs and career choices [43], graduate employment [41], and the quality of conference abstracts as a proxy indicator for knowledge and skills application [35]. The evaluation objectives of one study linked directly to the objectives of the FETP program [30].

The data collection methods included review of existing documents or data [34, 35, 39, 40, 42–45], knowledge test results [36, 37], surveys of fellows and/or graduates [30–32, 34, 36–38, 42], surveys of advisors and/or mentors [31, 42], surveys of FETP directors [14, 45], interviews with fellows and/or graduates [32, 36, 45], interviews with supervisors [45], stakeholders [30], and directors at training sites [45], and focus groups with supervisors and staff at training sites [30].

Most of the evaluations (n = 12, 75%) used predominantly quantitative analytical methods. Two evaluations (13%) used predominantly qualitative methods and two (13%) employed a mixed-methods approach. Of the 16 evaluations, 10 (63%) included output measures, 13 (81%) short- and medium-term outcome measures, and four (25%) longer term impact measures.

Attribution was rarely addressed in the evaluations; only four studies acknowledged this issue, indicating that it was not possible to solely attribute outcomes or impacts to the FETP [36, 38, 39, 45]. Some studies also noted other factors that may have influenced outcomes, including higher level surveillance reforms, other training programs attended by graduates, and the presence of enabling conditions [38, 39].

### Key output, outcomes, and impacts

Many of the summary findings were general and indicated that the program had achieved its objectives [34], prepared graduates to implement field epidemiology activities [31], improved the knowledge, skills and competencies of graduates [36, 38, 39], or served as a viable and effective method for improving the skills, knowledge and practices of the public health workforce [32]. Some evaluations provided summary statements highlighting the broader outcomes and impacts of the program, such as strengthening the capacity and provision of health services in the country [30], strengthening the epidemiology capacity of fellows, mentors and host sites [42], making substantial contributions to public health and leadership across local, state, and national levels, as well as in government, industry, academia, and non-governmental organisations [40], and contributing to improving the trust of the public and the international community in the work of the organization hosting the FETP [41, 44].

A summary of the outputs, outcomes and impacts from the published evaluations is shown in Table 2.

**Table 2:** Outputs, outcomes, and impacts from published FETP evaluations, 2003—2022

Outputs	• Employment in public health field following graduation [41, 43] • Presentations [34] and publications [34, 43] • Quality of abstracts submitted to an international conference [35] • Number of outbreak investigations [36] • Number of program evaluations [36] • Number of mobilisations [44] • Number of graduates/graduation rate [14, 36, 40]
Outcomes - individual	• Advancement in career or job promotion [14, 34] Increased confidence [30, 31] • Increased capacity to undertake research (30) and publish findings [30, 31] • Ability to use data for evidence-based decisions [30, 32, 34, 36, 45] • Improved ability to analyse data and use computer software [38, 45] • Improved knowledge and skills to perform field epidemiology tasks [30–32, 34, 36, 38, 39, 45] • Increased influence in the workplace [32] • Improved ability to prepare reports and papers [31, 38] • Improved leadership skills [32] • Improved communication skills [32]
Outcomes - institutional	• Increased public health capacity as graduates contribute to public health, often in positions of influence [14, 31, 32, 34, 36, 40, 42] • Strengthened surveillance systems – including improvements to data collection and reporting [31, 32, 36, 38, 39, 45], integrating private laboratories into the public health surveillance system [45], evaluating surveillance systems [36] • Improved outbreak investigation capacity [31, 36, 40, 42, 44, 45], and the ability to respond to communicable and non-communicable disease threats [32, 44] • Enhanced institutions’ ability to respond to public health emergencies and disasters [31, 44] • Graduates trained others and shared field epidemiology skills with broader workforce [30, 36, 38] and provided supportive supervision [38] • Contributed to the development of strong networks, collaborations and/or partnerships [30, 32, 36, 42] • Developed and improved health protocols, guidelines, and policies [31] • Enhanced capacity to conduct evaluations of health programs [36] • Improved data collection for reportable diseases [31, 32, 36, 38, 39, 45] • Development of advanced methodologies and analytical techniques [44]Improved active case finding [38, 44] • Graduates introduced innovations esource-num></record></Cite>3</electronic-resource-nu(30), fresh perspectives and enthusiasm [42] • Graduates acted as liaisons between government departments [40] • Weakened the laboratory sector as graduates moved into public health [14] • Ministry of Health failed to recognise full capacity of field epidemiologists and did not utilise them optimally [31] • Failed to utilise field epidemiologists for regional outbreak responses [31]
Impact	• Influenced national and state legislative and policy changes to protect public health [44] • Identified new infectious diseases during investigations and supported subsequent control measures [44] • Strengthened health systems by directly contributing to the national public health workforce and indirectly changing behaviours and skills in the wider workforce [30] • Increased public and international trust in national public health institution [44] • Greater visibility of field epi services in government and more broadly [30] • Increased government confidence in field epidemiology capacity [31]

## Discussion

FETPs aim to develop a cadre of health professionals capable of engaging in surveillance, responding to acute public health threats, and strengthening health systems based on scientific evidence [46]. This paper reviewed 16 published evaluations of FETPs from 2003 to 2022. Considering the large number of FETPs globally, and the importance of these programs in developing a national public health workforce, there are relatively few formal evaluations published. Those published are generally narrow in scope, rarely focused on impact and are of varying rigour. As a result, there is little direct evidence of the impacts attributable to FETPs.

While the intent of this article was to study the evaluation approaches and not to summarize the outcomes or impacts of FETP, the findings were overwhelmingly positive. Numerous outputs and outcomes described and quantified evidence of trainees and graduates applying skills to strengthen core health system functions. Several challenges were also identified, including poor utilisation of FETP graduates by senior management stemming from a poor understanding of what field epidemiologists can contribute to the health system. The evaluation of the laboratory track FETP highlighted alignment issues and unclear career pathways for graduates from these programs.

In addition to the published evaluations included in this paper, there is a body of literature describing the experiences and lessons learned from FETPs [11, 17, 47–56]. Although not formal evaluations, and not included in this review, the outputs listed, and the examples of program outcomes and impacts, highlight the influence of FETPs in responding to public health challenges around the world. For example, a review of 49 CDC-supported FETPs from 2005 to 2007 reported fellows conducting around 3,300 outbreak investigations during their training [7]. A similar paper reporting on outputs from 57 FETPs documented 4,663 outbreak investigations and 1,255 surveillance evaluations undertaken by fellows over four years from 2009 to 2012 [47]. Other papers provided specific examples of outbreaks investigated by fellows, including Ebola in West Africa (~ 70 graduates from 9 countries) [7, 57–59], SARS [8, 47], West Nile Virus [47], Typhoid [60, 61], Encephalopathy [62], Avian (H5N1) and swine (H1N1) influenza [47], Yellow Fever [47], Cholera [47, 56, 61, 63], Measles [47, 64], Rabies [47], Hepatitis A [61, 65], Middle East Respiratory Syndrome (MERS-Cov),[66] Marburg [48], Foodborne diseases [48, 61], and COVID-19 [67]. Fellows have also been involved in disaster response activities [47, 63, 68] and supported global public health mitigation and eradication initiatives, including Polio, Human Immunodeficiency Virus (HIV)/Acquired Immunodeficiency Syndrome (AIDS), and malaria [56, 65, 69–71]. Examples of specific outcomes and impacts from FETP fellows included the establishment of a surveillance and response system in the Philippines that resulted in a dramatic fall in fireworks-related injuries [61], improvements in vaccine coverage in Papua New Guinea [53, 61], repairs to and reconstruction of water systems following a cholera outbreak investigation in the Philippines [61], influencing national controls programs and policies for diseases such as Ebola, measles, Hepatitis B and HIV/AIDS in Uganda and Thailand [61], and changes to a national vaccination schedule following a rubella investigation [6]. Several studies referred to graduate retention in the public health workforce and career progression as indications of health system strengthening [7, 53, 64, 72, 73].

While these overview papers all point towards a successful training program, there are relatively few published evaluations providing the level of evidence increasingly expected by funders and stakeholders. Only four of the 16 publications identified in this review focused on assessing program impact [30, 31, 42, 44]. The tendency to focus on measuring outputs is a shared experience across the development sector, as they are relatively easy to measure [74]. However, given the high monetary and opportunity costs of FETPs, measuring impact and demonstrating value for money is essential to ensure ongoing investment. International development agencies are placing increasing emphasis on addressing development effectiveness and impact [75]. Demands for increased accountability have come not only from donors, but also from those impacted by programs who want to know how they and their communities will benefit following their engagement with development partners [76].

There were several different approaches used to evaluate FETPs, with the majority employing surveys or interviews to present descriptive reviews. The scope of the evaluations varied from evaluating specific segments of a single country-level program to broad multi-country program evaluations. The quality of the evaluations also varied. Several lacked specific evaluation objectives and none documented key evaluation questions. Most did not adequately address the issue of attribution or contribution. Only a few followed or referenced a specific evaluation model or theory. Evaluations rarely described negative outcomes or attempted to measure the indirect or unintended outcomes of a program.

Strengthening evaluations to address attribution is especially important. Attribution examines how much of the success (or failure) can be attributed to the program. There are almost always other factors at play that affect the outcomes of an FETP. Without assessing attribution, evaluators cannot know how much the FETP resulted in or contributed to the changes observed or consider how external factors influenced or prevented changes. Historically, the question of attribution was addressed using counterfactual assessments to estimate what would have happened without the program intervention [77, 78]. These experimental or quasi-experiment approaches are relatively complex requiring specialised expertise and considerable time and resources to undertake. However, with the rise of theory-based evaluation approaches, which do not require counterfactuals, simpler methods to address attribution have emerged. Contribution analysis is one such method and is well suited to FETP impact evaluations [79, 80]. Its aim is not to prove the effects of interventions, but to reduce uncertainty about their contribution to any changes that have occurred [80, 81].

There is a need and opportunity to develop tools and resources to support FETP evaluators, especially in the implementation of impact evaluations. The many different evaluation methods, models, and theories can be challenging to navigate. Many approaches are complex, requiring specialised expertise and/or extensive resources to implement. The FETP community would benefit from tools and resources that support evaluation approaches that are flexible, simple, and cost-effective. A one-size-fits-all approach using standard methods or tools does not cater to the diversity of FETP programs and the contexts within which they operate. Of greater value would be a FETP evaluation framework or implementation guide that supports evaluators in selecting, adapting, and applying methods and tools that focus on program outcomes and impacts. The overall approach to FETP evaluation should be based on international best practices. This includes the use of mixed methods approaches that are theory-based [77]. Theory-based approaches have the benefit of being simpler and more cost-effective than more traditional experimental designs. They also allow evaluators to assess direct and indirect causal contributions and include positive and negative, intended and unintended, direct and indirect, and primary and secondary effects resulting from an FETP [78, 82]. Any guidance tools, resources, frameworks or implementation guides should based on earlier guidance documents published by TEPHINET [26] and the CDC [46, 83].

This review is limited in that it focuses only on evaluations published in peer reviewed literature; grey literature and unindexed research were omitted. The authors are aware of unpublished and confidential FETP evaluations which were not included in this review. The extent of unpublished FETP evaluations is not known. It is possible that evaluations revealing unfavourable findings are more likely to go unpublished, resulting in publication bias. This review is also limited in that it did not include evaluations published in languages other than English.

## Conclusion

The development and expansion of FETPs over the past 70 years demonstrates the value and appeal of this applied work-integrated-learning training model. The outcomes and impacts described in published evaluations suggest program success and highlight the important role of FETPs in strengthening health systems and responding to disease threats. Despite the rapid global growth of FETPs, there have been relatively few formal evaluations, and even fewer focused on impact evaluation. The development of an FETP impact evaluation framework with accompanying tools and resources would greatly assist program evaluators and provide evidence of program impact. Such evidence will support programs in securing sustainable funding and will guide the ongoing implementation of FETPs around the world.

The development and expansion of FETPs over the past 70 years reflects the value of this applied, work-integrated learning model. The outcomes and impacts described in published evaluations suggest that FETPs are successful in strengthening health systems and building workforce capacity to respond to disease threats. Yet, despite global expansion, formal evaluations—especially those assessing impact—remain limited. A structured impact evaluation framework for evaluating FETPs, with supporting tools and resources, would enhance evaluation quality and provide much-needed evidence of program impact. This, in turn, would support continued investment and guide the ongoing development and implementation of FETPs worldwide.

## Data Availability

No data sets were generated or analysed during the current study.

## Appendix A

Search terms and strategy

**Table:**

**Date**	**Database Searched**	**Search strategy** (keywords, subjects, filters used)	**Relevant citations**
9 Jan, 2023	PubMed Title & Abstract	("Field Epidemiology Training"[Title/Abstract] OR "FETP"[Title/Abstract] OR "Applied Epidemiology"[Title/Abstract] OR "Epidemic Intelligence"[Title/Abstract]) AND ("evaluation"[Title/Abstract] OR "assess*"[Title/Abstract] OR "review*"[Title/Abstract])	Returned 143
9 Jan, 2023	DOAJ Abstract	("Field Epidemiology Training" OR "FETP" OR "Applied Epidemiology" OR "Epidemic Intelligence") AND ("evaluation" OR "assess*" OR "review*)	Returned 22
9 Jan, 2023	Scopus Title, Abstract, Keywords	TITLE-ABS-KEY ( ( fetp OR "Field Epidemiology Training" OR “Applied Epidemiology” OR “Epidemic Intelligence”) AND evaluation OR assess* OR review*)	Returned 237
